# In flight fragmentation reduces bomb size range and hazard during explosive volcanic eruptions

**DOI:** 10.1038/s41598-025-20900-2

**Published:** 2025-10-22

**Authors:** C. Biensan, J. Taddeucci, M. Alatorre-Ibarguengoitia, P. Scarlato, D. Andronico, T. Ricci, E. Del Bello, L. D’Auria, M. Asensio-Ramos, D. M. Palladino

**Affiliations:** 1 Istituto Nazionale di Geofisica e Vulcanologia, Sezione Roma 1, Via di Vigna Murata 605, 00143 Roma, Italy; 2 Instituto de Investigación en Gestión de Riesgo y Cambio Climático, Universidad de Ciencias y Artes de Chiapas, Libramiento Norte poniente 1150, Lajas Maciel, 29039 Tuxtla Gutiérrez, CHIS Mexico; 3 Istituto Nazionale di Geofisica e Vulcanologia, Osservatorio Etneo, Piazza Roma 2, 95125 Catania, Italy; 4 Instituto Tecnológico y de Energías Renovables (ITER), 38600 Granadilla de Abona, Tenerife, Canary Islands Spain; 5 Instituto Volcanológico de Canarias (INVOLCAN), 38320 San Cristóbal de La Laguna, Tenerife, Canary Islands Spain; 6 Dipartimento di Scienze della Terra, Sapienza-Università di Roma, Piazzale Aldo Moro 5, 00185 Rome, Italy

**Keywords:** Solid Earth sciences, Volcanology

## Abstract

Coarse, molten fragments of low-viscosity magma (volcanic bombs) that are ejected during explosive volcanic eruptions represent a source of hazard and a record of past eruptions. After ejection, bombs tend to break up during flight, but how much this affects their dispersal is unclear. Here, we use high-speed and high-definition imaging of three recent explosive eruptions to parameterise the in-flight fragmentation of bombs. We estimate that in-flight fragmentation involves 73% of bombs coarser than $\sim$ 0.2 m, with bomb-to-bomb collisions and aerodynamic frictional (drag) forces being the main drivers of in-flight fragmentation, depending on eruption style. Drag force increases with increasing bomb velocity and size, selectively fragmenting the coarsest and fastest bombs, acting as a self-limiting factor for the range and energy of falling bombs. These findings pose a quantitative basis for incorporating the in-flight fragmentation processes into the interpretation of volcanic deposits and for modelling hazards from falling bombs.

**Supplementary Information:**

The online version contains supplementary material available at 10.1038/s41598-025-20900-2.

## Introduction

Volcanic bombs are coarse (> 64 mm), variably molten fragments of low-viscosity magma ejected during explosive volcanic eruptions. Volcanic bombs travel along approximately ballistic trajectories, mainly controlled by the ejection velocity and angle acquired at the vent and friction with the surrounding fluid (volcanic gas jet or surrounding atmosphere)^1–5^. Ejection angle and velocity being the same, coarser bombs will travel further due to their higher mass-to-surface ratio and corresponding higher inertia-to-friction ratio. In the eruption of relatively hot and low-viscosity mafic magmas, such as basalt, bombs remain mostly fluid during their ballistic transport. This increases the potential for in-flight deformation and fragmentation to alter bomb size, trajectory, and, eventually, the associated hazards and deposit features^5–11^. Despite its relevance, however, in-flight bomb break-up is poorly described qualitatively and quantitatively, with a few exceptions^5,12^.

The fallout of volcanic bombs represents one of the most significant hazards in the range of a few kilometres from the vent of explosively erupting volcanoes, often associated with small and unexpected eruptions^13–15^. It affects both visiting tourists and scientists conducting measurements in high-risk zones, with examples from Stromboli and Etna (Italy) and Yasur (Vanuatu) volcanoes^16–19^. Bombs have been documented to reach distances of 2–5 km from the vent, and up to 10 km^7,13,17,20–22^ Bombs also pose a significant economic threat, damaging infrastructure and buildings^14,17,23,24^.

Field- and drone-based studies on the dispersal and grain-size distribution of bomb deposits are crucial to understand the dynamics of past eruptions and the definition of hazard maps^7,12,19,22,25–31^. Ballistic dispersion modelling is often used for hazard forecast and to infer exit velocity during past explosive eruptions^2,4,26,32–34^. Up to now, however, neither modelling or deposit interpretation schemes take into account in-flight bomb fragmentation. Here, we analysed high-speed and high-definition videos of three recent explosive eruptions to qualitatively and quantitatively characterise the in-flight fragmentation of fluidal bombs.

### Case studies

**Fig. 1:**
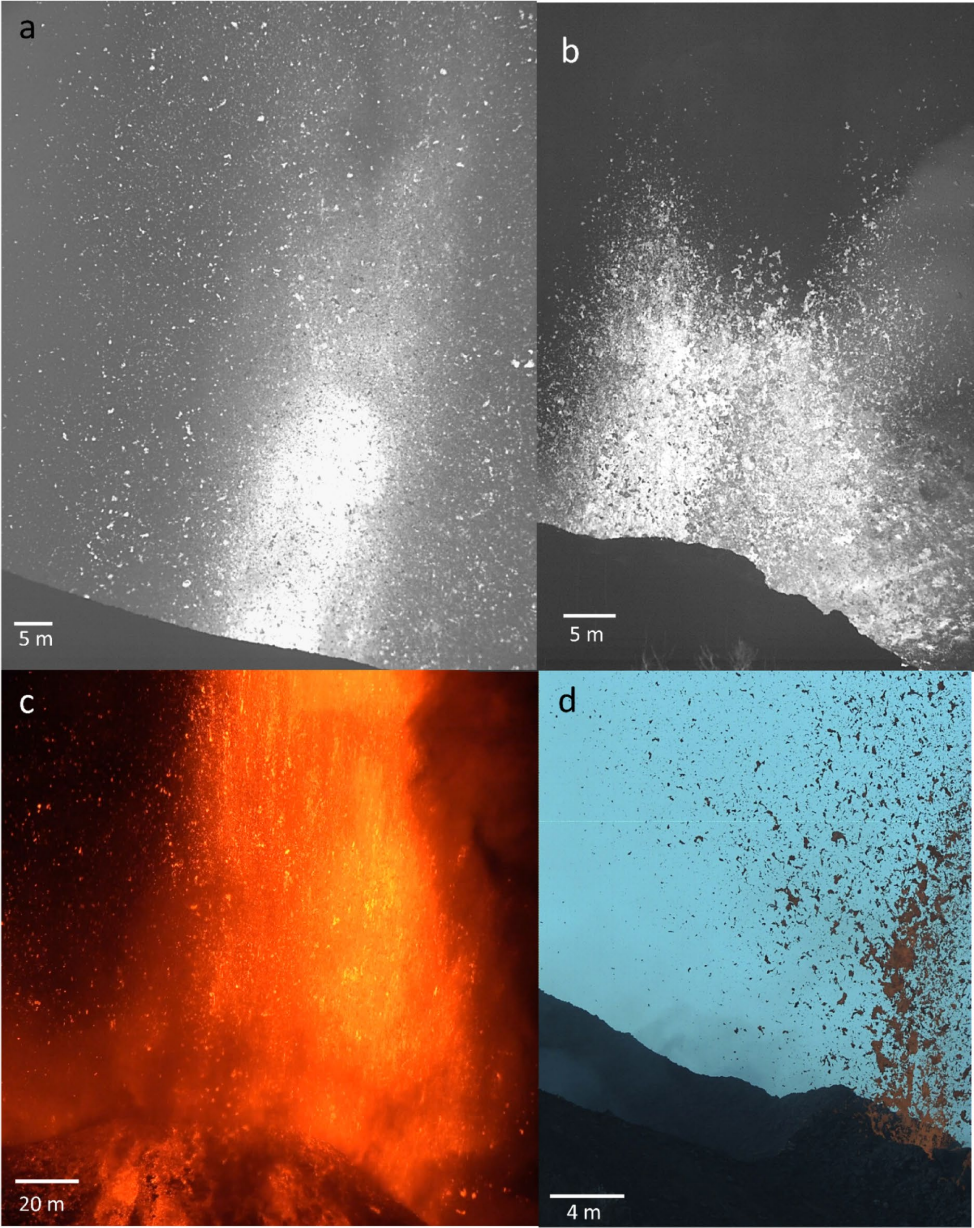
Explosive eruption case studies. Cropped still frames from the original videos used for the analysis of in-flight bomb fragmentation. (**a**) Fountaining during the 2021 Tajogaite eruption of the Cumbre Vieja volcanic system, La Palma, Canary Islands (Spain), (**b**) Spattering (also termed low fountaining) during the 2021 Tajogaite eruption. (**c**) Fountaining during Mount Etna (Italy) eruption in 2021. (**d**) Strombolian activity at Stromboli Island, (Italy), in 2023. In the greyscale images hotter bombs are in brighter tones (see Supplementary Movies 1–4).

To cover a range of volcanoes and styles in low-viscosity magma explosive activity, four case studies are the focus of this inquiry (Fig. 1). The first two case studies are the fountaining activity (Fig. 1a) and the spattering activity (Fig. 1b) of the eruption of the Tajogaite volcano (19 September to 13 December 2021), part of the Cumbre Vieja volcanic system (La Palma, Canary Islands, Spain). The activity of this eruption fluctuated significantly, exhibiting a wide range of eruption styles including fountaining, spattering, ash-rich jets, and ash-poor jets, alongside persistent emission of lava flows^35–38^. Bombs have a tephrite/basanite bulk composition^39,40^. Fountaining involved the ejection of metre-sized bombs, lapilli, and varying amounts of ash, with exit velocity up to 210 m/s^39^ and the formation of up to kilometre-high plumes^41^. Fountaining lasted hours to days and exhibited pulsating ejections on a second-to-second basis. Spattering, or low fountaining, ejected decimetre- to metre-sized fluidal bombs at heights between 50 and 200 m and velocity up to 55 m/s, without ash plume formation. Also spattering was characterised by discrete pulses on a second-to-second scale^38^.

The third case study is the 24 February 2021 lava fountain episode of the South-East Crater of Mount Etna (Fig. 1c). During the paroxysmal activity, jets of incandescent bombs and lapilli reached 500 m of height topped by an eruption column of 11 kilometres height at a maximum, with ash and lapilli spread up to tens of kilometres from the vent. The lava fountaining activity erupted trachybasaltic magma^42^ and lasted just over 2 hours. The ejection velocity reached up to 170 m/s with a pulsating behaviour on a second-to-second basis (this study).

The fourth case study is the ordinary strombolian activity at Stromboli volcano (Fig. 1d), characterised by minutes-spaced discrete explosions that propel bombs to heights ranging from 100 to 200 m above various vents, with explosions lasting from seconds to tens of seconds and with exit velocity of up to 400 m/s^5,43–45^. Its magma composition is shoshonite^46^. We focus on the activity of the North-East vent area on 22 October 2023, which produced ash-poor explosions.

Hereafter, we refer to the four case studies as T. for Tajogaite, E. for Etna and S. for Stromboli followed by the eruptive style: (i) T. fountaining, (ii) T. spattering, (iii) E. fountaining, and (iv) S. strombolian.

## Results

### Modes of in-flight fragmentation

**Fig. 2:**
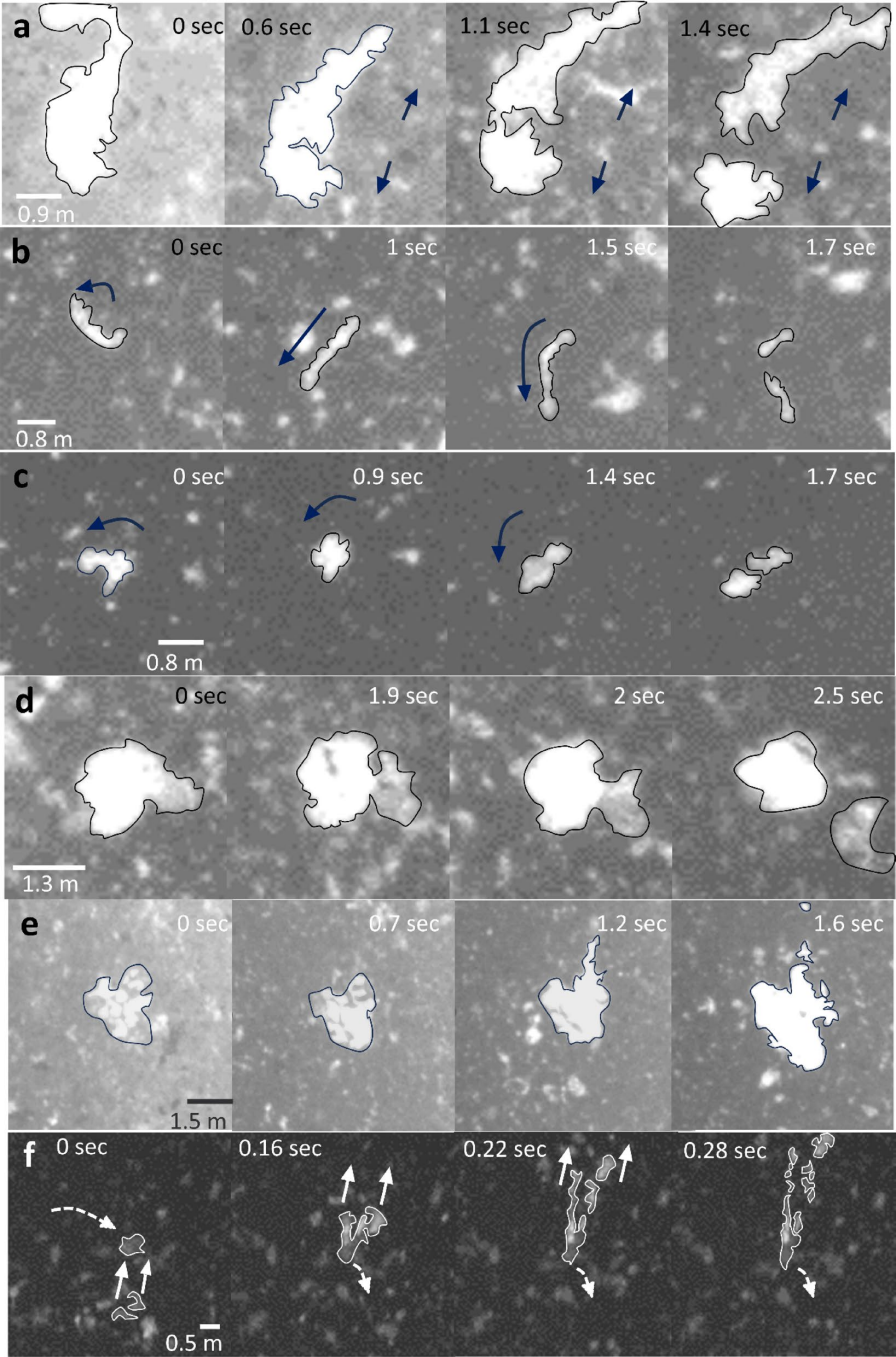
Modes of in-flight fragmentation of bombs. Still frames depicting the time evolution of the various fragmentation modes. (**a**) Deformation fragmentation by stretching of a bomb. Stretching, thinning of a narrow bridge, and final break-up occur in several parts of this bomb in less than one second (Supplementary Movie 7). (**b**) Continuous bending leading to localised stretching and final fragmentation of a high-aspect ratio bomb (Supplementary Movie 11). (**c**) Rotation causing a bilobate bomb to stretch and eventually fragment at the centre (Supplementary Movie 15). (**d**) Detaching fragmentation. The colder (darker) part of a bomb detaches and drags away portions of the hotter part (Supplementary Movie 19). (**e**) Inflating fragmentation of a bomb. The visible outer layer of the bomb has initially variable temperature (note brighter spots) and is deforming. Collision with a rising bomb (at time 1.2 s) triggers sudden expansion, breaking of the colder outer layer, and runaway fragmentation (Supplementary Movie 21). (**f**) Collision fragmentation of two finer rising bombs with a coarser, falling bomb. The collision produces multiple, hot (bright), rising fragments and causes stretching and then breaking (not visible in the figure, but visible in Supplementary Movie 25) of the falling bomb. All examples are taken from the T. fountaining case, except (**f**) that is taken from the T. spattering case. Downwards is at the bottom in all images. Brighter tones indicate higher temperature (See Supplementary Movies 5–25).

From the visual analysis of the videos we defined four distinct modes of in-flight fragmentation (Fig. 2, Supplementary Movies 5–25):

i. Deformation fragmentation, in which bombs visibly deform before starting to fragment and finally break at their thinnest visible point. This fragmentation mode encompasses three mutually non-exclusive variants: (1) stretching (Fig. 2a, Supplementary Movies 5–8), in which bombs exhibit elongation with sizes ranging from centimetres to metres, and may vary in brightness (function of surface temperature), (2) bending (Fig. 2b, Supplementary Movies 9–12), typical of elongate and sometimes bilobate bombs^5^ and (3) rotating (Fig. 2c, Supplementary Movies 13–16), most common for bilobate bombs.
ii. Detaching fragmentation (Fig. 2d, Supplementary Movies 17–19), when no visible deformation precedes the separation of the bomb into fragments.
iii. Inflating fragmentation (Fig. 2e, Supplementary Movies 20–21), where mostly metre-sized bombs fragment after expansion. The exterior of the bomb appears darker because of cooling during aerial transit and, on expansion, gradually breaks, exposing the hotter and brighter interior core. Expansion is followed by shredding and fragmenting of the hotter parts.
iv. Collision fragmentation (Fig. 2f, Supplementary Movies 22–25), involves the collision between two or more bombs travelling at different velocities and often with different direction and size. Breaking upon collision very often causes an increase in the brightness of bombs, as the external darker crust breaks and reveals the hotter, brighter inside.

In several cases single bombs fragment with more than one mode in a short time interval (Supplementary Movies 21–22). On average, fragmentation by deformation and collision modes results in the generation of about two and six fragments, respectively. In the deformation mode, bombs fragment 47% in fall, 48% while rising, and 5% close to the top of their trajectory.

### Quantification of in-flight fragmentation

**Fig. 3:**
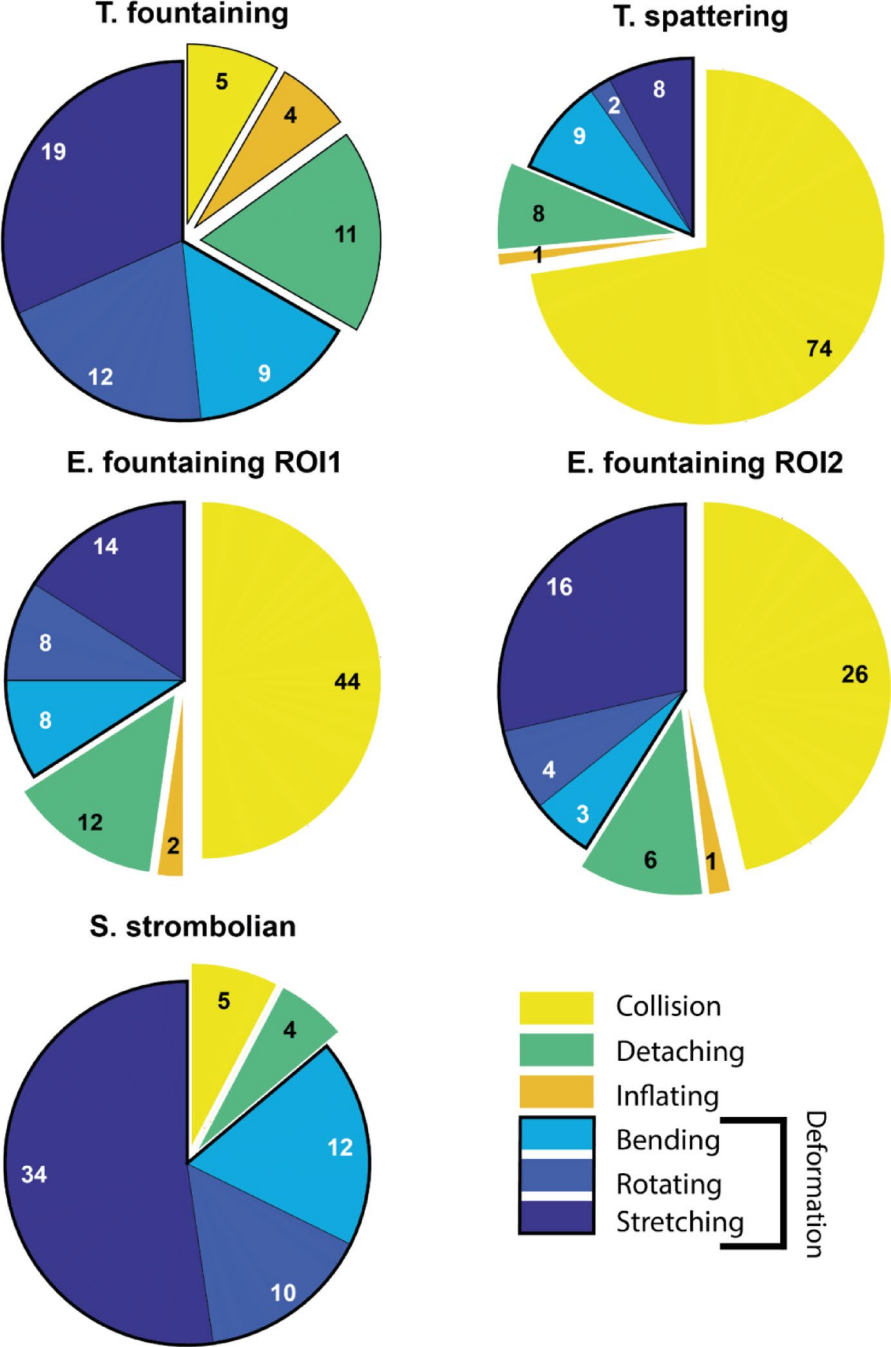
Incidence of in-flight fragmentation modes in the different case studies. Relative abundance (size of pie slice) and number (number in the pie) of observed in-flight fragmentation events for the different fragmentation modes in the four case studies (note the two ROIs for the E. fountaining case). A black, thicker border groups the different variants of the fragmentation by deformation mode.

The relative occurrence of in-flight fragmentation can be estimated by comparing the number of fragmenting bombs over the total number of bombs transiting the analysed Region Of Interest of the videos (ROI) in the observed time interval and within the same size range, which is limited by the size range of the observed fragmenting bombs. The fragmenting bomb population has been carefully measured visually (see Methods). To assess the total bomb population, we used an automatic detection algorithm and compared its results with the total number of bombs detected manually in one video (see Methods and Supplementary Information). The percentage of in-flight fragmenting bombs over total bombs within the same size range varies significantly in the four case studies, ranging from 12 to 73%, with an average over all cases of 37% (Table 1 and Fig. 3).

**Table 1:** Total fragmenting bombs and fragmentation per cent.

Case study	Total bombs* (#)	Size range (m)	Fragmenting (non-collision, collision) (#)	Fragmenting (non-collision, collision) (%)	Total fragmenting (%)
T. fountaining	490	0.24–1.38	55, 5	11, 1	12
T. spattering	140	0.27–0.97	28, 74	20, 53	73
E. fountaining (ROI1)	199	0.57–2.47	44, 44	22, 22	44
E. fountaining (ROI2)	173	0.72–2.32	30, 26	17, 15	32
S. strombolian	122	0.14–1.14	60, 5	49, 4	53

* Average of the total number of bombs detected over 5 non-consecutive frames within the appropriate size range, multiplied by 0.62, i.e., the fraction of manually detected bombs versus automatically identified ones in the T. fountaining case (see Methods).

Deformation and collision are the two dominant modes of in-flight fragmentation, deformation dominating the T. fountaining and the S. strombolian cases, and collision prevailing in the T. spattering and, to a lesser degree, the E. fountaining cases (Fig. 3). Within the deformation mode, stretching slightly prevails but there is quite a variability in between the different cases. Detaching fragmentation is largely subordinate but still present in all cases, and inflating is rare, with a maximum of four occurrences (T. fountaining). We note that, despite utmost care was followed in the visual classification, uncertainty in the attribution of inflating and detaching modes remains. The results for the two Etna ROIs show that for the ROI2, located near the vent, there is more fragmentation via stretching and less via collision than on the ROI1, located further away from the vent, but overall the results are consistent, suggesting no major differences in in-flight fragmentation in the two zones of the eruption (Fig. 3).

### Bomb properties in collision-induced fragmentation

**Fig. 4:**
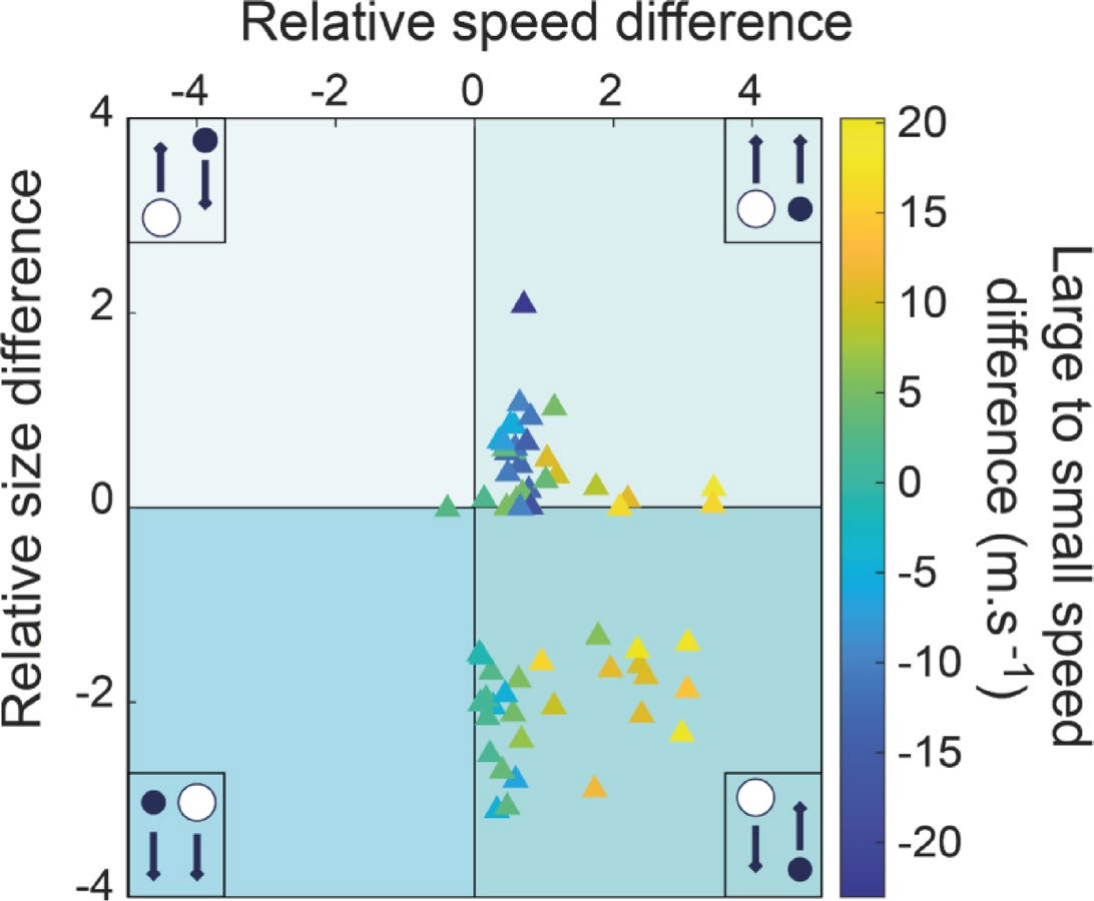
Size, velocity, and direction of bombs undergoing collision-induced fragmentation. Limited to the T. spattering case, the four quadrants represent all possible size-direction combinations of colliding pairs (see insets). Relative speed and size differences (dimensionless) are the speed and size difference (finer minus coarser) between bombs divided by the speed or size of the finer bomb, respectively. Colour scale values indicate the speed difference between the larger and finer bombs, and are negative (blue) or positive (yellow) if the faster bomb is the finer or the coarser, respectively.

The T. spattering case stands out with a substantial 53% occurrence of collision-induced, in-flight fragmentation and offers a rich opportunity for focused investigation. We measured separately the size, direction, and speed of the two bombs in each colliding pair, calculating the relative size and speed difference. This has been done by dividing the size and speed difference (size and speed of the coarser bomb minus the size and speed of the finer one) by the size and speed of the finer one. We assigned positive or negative values of size and velocity to rising and falling bombs, respectively. Almost all collisions (92%) occurred with the finer bomb rising, equally divided between rising (46%) and falling (46%) coarser bombs. Rarely (2%) bombs collided while both were falling, and no fragmentation-inducing collision was observed when the finer bomb was falling (Fig. 4). Relative size and speed differences range from − 3.1 to 2 and from − 0.4 to 3.4, respectively. When both bombs were rising, collision-induced fragmentation occurred due to speed or size differential, but not a mix of the two. Conversely, when the coarser bomb was falling and the finer rising, fragmentation occurred because of a mix of relative speed and size differences (Fig. 4). In general, with increasing relative speed difference, the faster bomb shifts from being the finer to being the coarser one. No agglutination of colliding bombs has been observed.

### Bomb properties for deformation-induced fragmentation

To understand the factors that control deformation-induced fragmentation, we compared the size, shape and velocity distribution of fragmenting and total bomb populations, here not limited to the same size range but within the broadest range of measurable bombs. The absolute value of velocity (independent of rising or falling direction) was used and collisions were ignored. All measured parameters refer to bombs before they fragment.

**Fig. 5:**
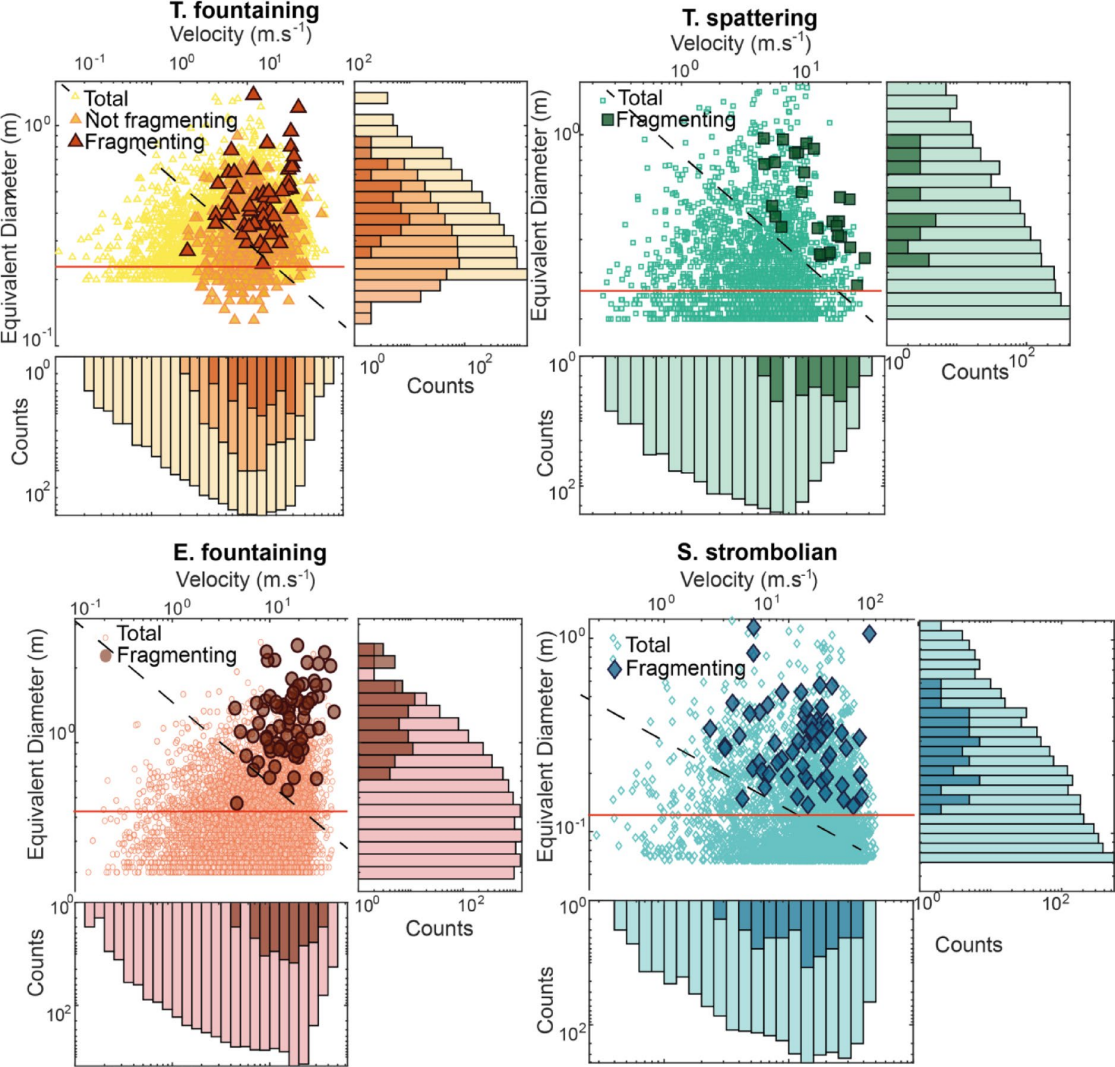
Size and velocity of total and in-flight fragmenting bomb populations, collisions excluded. Bomb equivalent diameter versus velocity for total (lighter color in the histograms) and in-flight (darker colors) fragmenting bombs for the four case studies, excluding fragmentation by collision. The T. fountaining case also includes manually detected, non-fragmenting bombs. Dashed lines mark approximately the size-velocity threshold for the appearance of in-flight fragmentation. Note logarithmic axis scales variable from one case to another. Error on size is $\pm$ one pixel (see Table 2) and 2.1 m/s for the E. fountaining case and 1.1 m/s for all other cases. The truncated size distributions of the total population reflect the detection threshold of the automated algorithm. Bombs below the red horizontal line were discarded from the calculation of the fraction of fragmenting bombs.

The distributions of bomb size, circularity, and velocity vary significantly across different case studies, depending on eruption style, selected ROI, video resolution, and eruption time relative to ejection pulses. Summing up all case studies, total bombs range from 0.1 to 2.6 m in size and from 0 to 64 m/s in velocity. In-flight fragmenting bombs have a similar size range from 0.1 to 2.5 m, but a higher velocity range from 2 to 68 m/s (Fig. 5). For the total bomb population, in the T. fountaining case (Fig. 5), non-fragmenting bombs detected manually and total bombs detected automatically display an average size of 0.3 m and average velocity of 13 m/s and 12 m/s, respectively, supporting the reliability of the automated method for measuring bombs (see Methods). Both methods reveal a unimodal distribution of size and velocity, like the pattern observed in the E. fountaining case, which have a mean size and velocity of 0.5 m and 11 m/s (Fig. 5), respectively. The T. spattering case exhibits a unimodal distribution of bomb size (average 0.35 m) and velocity (average 5 m/s) (Fig. 5). The S. strombolian case features bombs that are generally finer grained (average 0.14 m) but exhibit relatively high velocities (reaching 50 m/s). We note that all size distributions from the automated detections are truncated towards the finer values, due to the video resolution and the detection threshold we imposed (see Methods). The velocity measurements, conversely, seem to capture most of the distribution (Fig. 5).

For the fragmenting bomb population, bombs from the E. fountaining case show a notably broader size range, averaging 1.31 m, compared to a maximum of 1–1.4 m observed in other eruptions. The finest fragmenting bombs, 0.07 m in size, were observed in the S. strombolian, while the coarsest, reaching up to 2.5 m, were identified in the E. fountaining case. Velocity distribution varies also significantly: the lowest velocities were recorded for S. strombolian and T. fountaining case, with a minimum of 0 m/s, whereas the highest velocities, peaking at 40.3 m/s, were observed in the S. strombolian case.

The modal values of the size and velocity distributions are higher for the in-flight fragmenting population with respect to the total population, and, overall, the proportion of in-flight fragmentation events increases with increasing size, velocity and circularity.

**Fig. 6:**
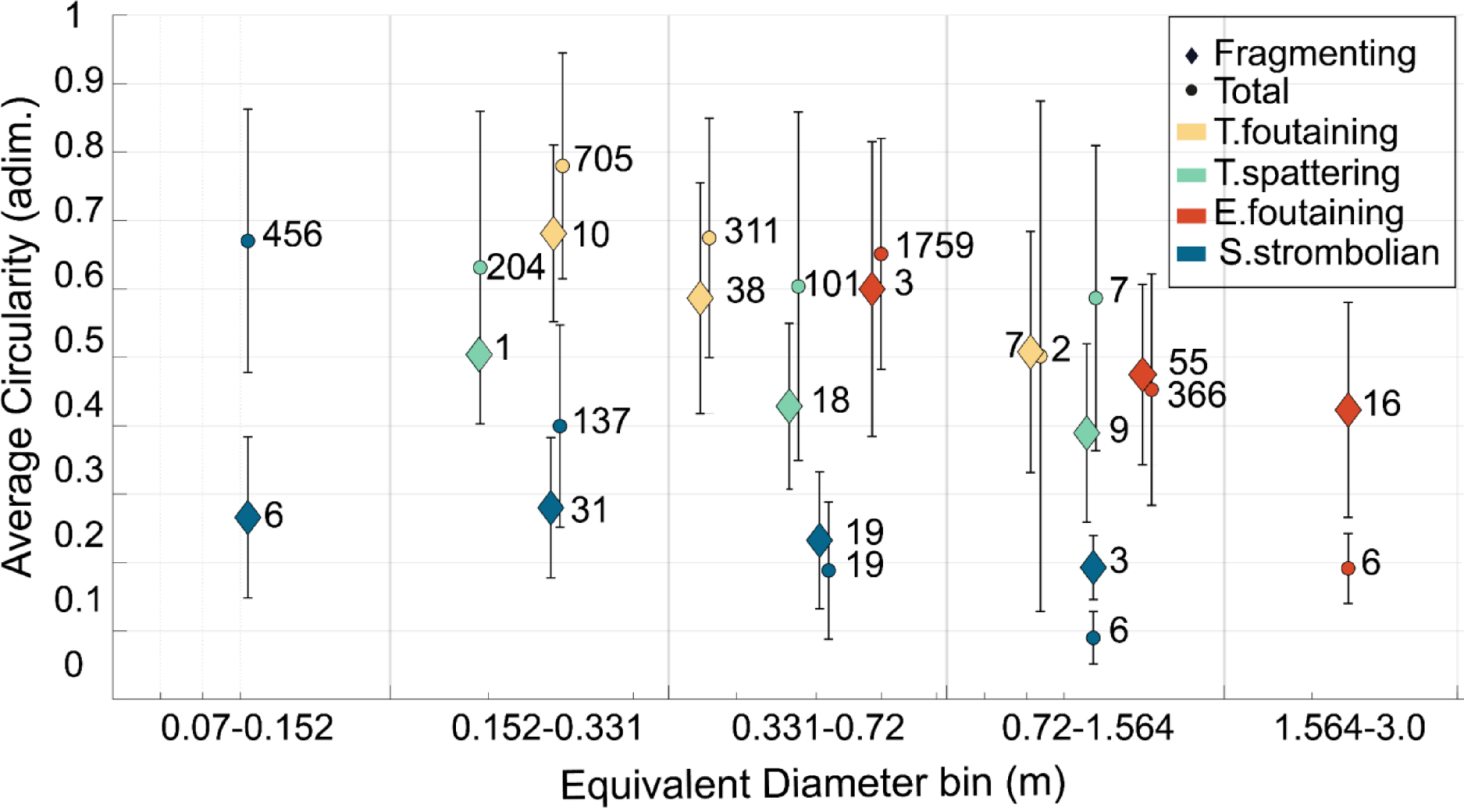
Circularity versus size of total and in-flight fragmenting bombs, collisions excluded. Average circularity as a function of binned size. Higher circularity values indicate more regular shape. Numbers in the plot are the number of bombs in the size bin. Error bar is the standard deviation of the circularity per each case.

Overall, bomb circularity decreases with increasing size, as recently found at Stromboli and Etna^47^. Fragmenting bombs of both Tajogaite cases consistently exhibit slightly more irregular shapes than the rest. The S. strombolian bombs have the lowest circularity, fragmenting ones being significantly more irregular than non-fragmenting ones in the smallest size bin. Conversely, in the largest size bin, both the S. strombolian and the E. fountaining fragmenting bombs tend to be slightly more regular in shape than the rest, but still have comparatively low circularity values (Fig. 6).

### Causes and mechanisms of in-flight fragmentation

This study reveals a diversity of in-flight fragmentation mechanisms for fluidal bombs across various eruptive styles, including fountaining, spattering, and strombolian activity. We identify several factors that variably control the different fragmentation mechanisms, including velocity, size, and shape of bombs and, subordinately, bomb location with respect to the vent, and development of the eruption through ejection pulses.

### Fragmentation by collision

Overall, collisions are the second dominant in-flight fragmentation mode, representing 32% of all observed in-flight fragmentation events, and exceeding 70% for the T. spattering case (Fig. 3), due to the dominantly vertical ejection, small spreading angle, and relatively small dispersal of bombs. Collisions can cause both brittle and ductile fragmentation of bombs^5,48^, but the latter case largely dominates our observations on low-viscosity magmas. For these magmas, theoretical estimates set the minimum velocity differential for collisional fragmentation at 20 m/s^48^, but we observe fragmentation at velocity differentials as low as 2 m/s ca.

Collisions occur due to differences in the velocity and trajectories of bombs, which are maximum at the onset of the individual ejection pulses that characterise all our case studies and similar eruptions^5,9,10,33,38,49–51^. In fact, of all the possible combinations of colliding bomb size, velocity and direction, only the two linked to ejection pulses were observed (Fig. 4): (i) fragmentation-inducing collision of bombs from two successive pulses, with the finer (usually faster) bombs released at the start of a later pulse reaching and impacting the slower (usually coarser) ones released at the tail of the preceding pulse; and (ii) the finer bombs released at the start of one pulse colliding and fragmenting the falling, coarser ones of the previous pulse. This dynamic is favoured by the larger dispersal of finer bombs with respect to the coarser ones, which fall closer to the vent where the rising ones are released. For the same reason, no collisional fragmentation of falling finer bombs has been observed. Finally, collision-induced fragmentation between two falling bombs is extremely rare, likely due to the relatively low velocity differential and larger dispersal of falling bombs. These results provide an empirical scheme for the likeliness of collision-induced in-flight fragmentation.

### Fragmentation by inflation

Fragmentation by inflation is rarely observed and represents only 5% of the in-flight fragmenting bombs in our cases at maximum. Inflation is likely driven by bubble expansion due to continued degassing, causing the rupture of the cooler, outer part of a bomb and exposing the hotter, inner core. Fluidal bombs frequently exhibit poorly vesicular outer rinds and/or greatly inflated interiors, suggesting quick external quenching concurrent with internal expansion. Namiki et al.^48^ suggest that adiabatic cooling of the gas and the rapid cooling of the external crust can lead to brittle fragmentation and the formation of finer particles. In our case, it appears that fragmentation induced by expansion is dominated by the drag of air on the protruded, hotter, and less viscous portions of the bomb that is inflating. In addition, the increased vesicularity weakens the bomb, thus favouring fragmentation^52^. Fragmentation by inflation has been observed only for the coarsest bombs, exceeding a metre in size, which cool more slowly, allowing more time for degassing.

### Fragmentation by detachment

Fragmentation by detachment is rare, representing only 1–15% of in-flight fragmentation events. The lack of visible deformation before fragmentation suggests a brittle fragmentation process occurring at the large (clast) scale. This may be due to stress accumulation below the glass transition temperature, although this seems hard in our low-viscosity, high-temperature case studies. Fracturing could locally be favoured by uneven thermal contraction, vesiculation, and crystallisation.

### Fragmentation by deformation

Fragmentation preceded by visible deformation of the bomb always represents more than 15% of in-flight fragmentation and between 60 and 80% in the T. fountaining and the S. strombolian cases (Fig. 3). The deformation fragmentation mode resembles the ductile, or inertial, fragmentation already recognised in low-viscosity magma^8,53–57^. Critical to this fragmentation is the presence of velocity gradients within a bomb. Velocity gradients favour fragmentation by inducing deformation that localises stress and strain rates at weak points^58–60^, which depend on the heterogeneities in the internal physical properties of the bomb, including temperature, vesiculation, and crystallisation^52,54,56,61–63^. A combination of these effects can locally bring magma at thin necks to localised brittle fragmentation^63^, as observed microscopically in all volcanic particles of basaltic and similar compositions^65^.

**Fig. 7:**
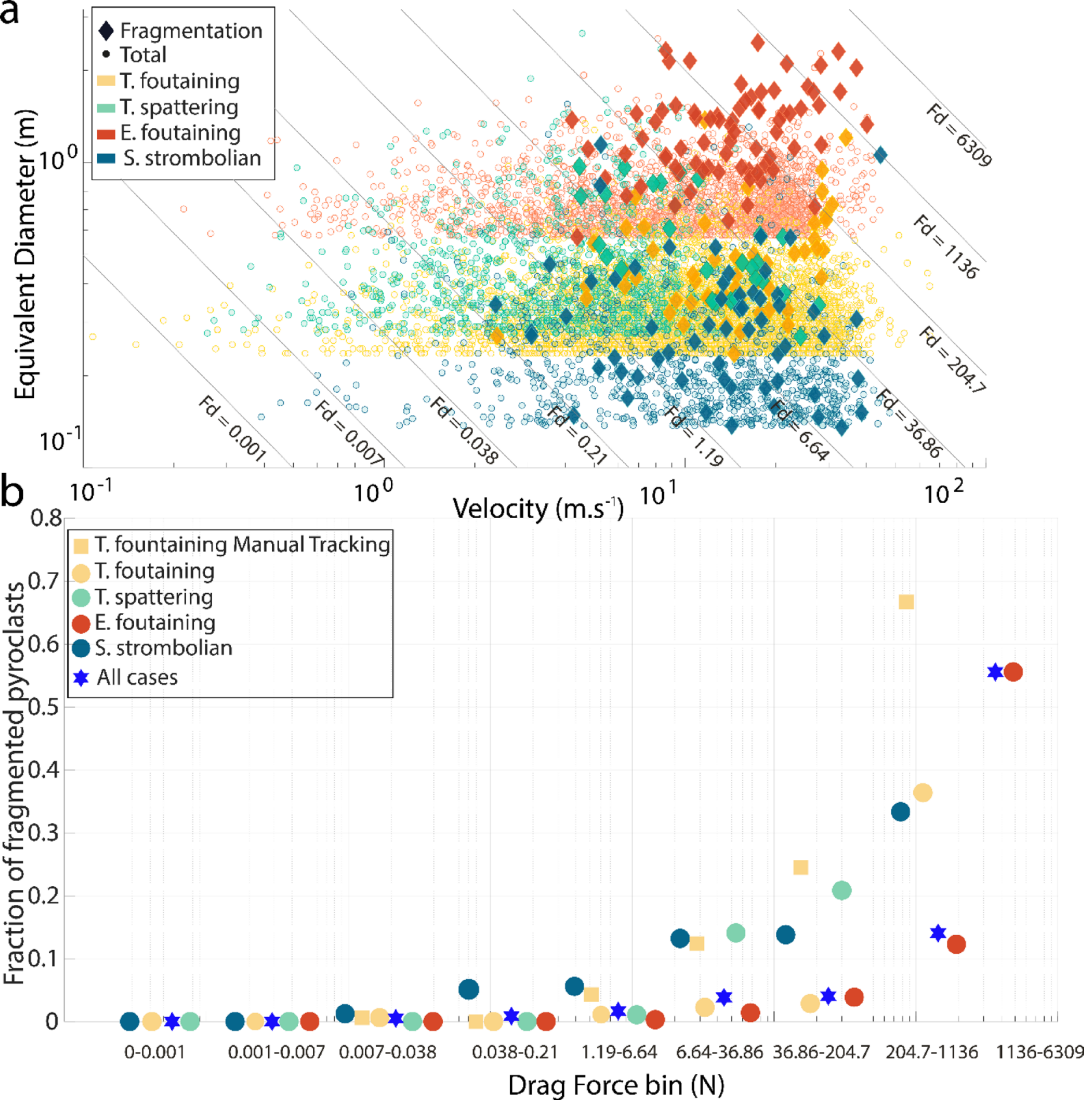
Relationship between bomb size, velocity, drag force, and in-flight fragmentation. (**a**) Equivalent diameter and velocity for total and in-flight fragmenting bomb populations, excluding collisions. Lines represent equal values of drag force (*F*_*D*_) for size-velocity combinations, computed assuming a drag coefficient (*C*_*d*_) equal to one. Error is as per Fig. 5. (**b**) Binned values of the drag force on bombs (with bin edges corresponding to the lines in (a)) versus the fraction of in-flight fragmenting bombs over the total number of bombs in the bin. Study cases are plotted individually and collectively. For the T. fountaining case the manual tracking results are also plotted.

Velocity gradients can originate at the vent, when the last part of the bomb to detach from the conduit or the magma moves slower than the rest of it, by collisions with other bombs, by the interaction of the bomb with volcanic gas jets, and by centrifugal effects^5,56,66^. Another cause for velocity differentials is the drag force, i.e., air resistance acting differently upon parts of the same bomb with different exposure to air flow or surface roughness, eventually causing fluid-dynamic instabilities. Drag may increase existing velocity gradients and generate new ones. The fact that coarser, faster, and more irregularly-shaped bombs are more subject to in-flight fragmentation (Figs. 5 and 6), supports a pivotal role of drag force. A unified plot of bomb size versus velocity for all case studies, limited to the size range of the fragmenting bombs and excluding collision-induced fragmentation, supports this hypothesis (Fig. 7). In a size-velocity space, it is possible to draw lines of equal drag force (*F*_*D*_) using Eq. 1 and taking for simplicity a fixed value for the drag coefficient *C*_*d*_ = 1, commonly used for bombs^5^ (Fig. 7a). Changing the assumed value of *C*_*d*_ would result in different values of *F*_*D*_ but would just shift the lines parallel to themselves. The occurrence of in-flight fragmentation increases in parallel with increasing *F*_*D*_. The effect of *F*_*D*_ on bomb in-flight fragmentation is even more evident if we calculate the drag force of all bombs and plot the fraction of fragmenting to total bombs in logarithmic drag force bins (Fig. 7b). The drag force *F*_*D*_, function of bomb size and velocity, is calculated as:

1 $$F_{D} = \frac{1}{2}\rho \nu^{2} C_{d} A$$

where $\rho$ is the air density (1.293 kg/m^3^), $\nu$ (m/s) is the measured bomb velocity, *Cd* is the drag coefficient, assumed constant and equal to 1, and $A$ (m^2^) is the bomb cross-sectional area, determined using the equivalent diameter of the bomb. The fraction of fragmenting bombs is zero for *F*_*D*_ ≲ 0.2 N, then rises, for all case studies, initially slowly as drag force increases and then rapidly for *F*_*D*_ ≳ 20 N. This simple modeling does not account for the more irregular shape of larger bombs, which would further increase the *F*_*D*_ of most fragmenting bombs.

The combined data from the four case studies define a common trend of increasing fragmentation fraction with increasing *F*_*D*_ which, in effect, is a drag force-controlled criterion for in-flight bomb fragmentation. A stronger drag force means stronger velocity gradients and strain differentials acting within a bomb. In other words, increasing the size and velocity of bombs increases, other physical parameters being the same, the ratio of inertial to other, i.e., viscous and capillary, forces^55,64^, as visible in the dimensionless analysis of our results (see Supplementary Information). It is worth noting that the four case studies define a common trend despite differences in observed area of the eruption (i.e., ROI), eruption style, magma physico-chemical properties. The Tajogaite melt fraction of magma, for instance, has an expected viscosity at eruption almost ten times lower than that of Etna and Stromboli^67,68^, but no gap separates in-flight fragmentation events of the four cases (Fig. 7). The common trend, then, suggests that bulk magma properties at eruption are relatively unimportant with respect to bomb properties at fragmentation. Viscosity, for instance, is expected to rise quickly as bombs cool in-flight. It may even vary strongly laterally within the same bomb, a function of local air flow and bomb thickness, as visible in our videos (Supplementary Movies 7, 17, and 21). Coarser bombs cool slower than finer ones, and their lower viscosity may combine with higher drag force in leading them to preferential in-flight fragmentation.

### Implications for bomb dispersal: hazards and deposits

This study has provided a field-based analysis of the in-flight fragmentation of fluidal bombs coarser than about 0.1 m across different eruptive contexts, and a first quantification of the several processes that cause it, dominantly air drag and collisions. Unquantified effects that also affect in-flight fragmentation include the development of eruptions through individual ejection pulses and the size-dependent cooling rate of bombs. Our results apply to all low-viscosity magmas with a composition similar to the case studies. These magmas are pervasive on Earth and other planetary bodies, and our results have implications impacting deposit interpretation, hazard mitigation and potentially planetary volcanism. In-flight fragmentation may also involve bombs smaller than those we analysed, but their lower size and range intrinsically reduce the hazard they pose.

An important consequence of the dominant effect of drag force is that in-flight fragmentation is more efficient for coarser and faster bombs. These are those that at a given ejection velocity would travel the longest distance following ballistic trajectories and would also pose the highest hazard on landing due to their higher kinetic energy. The finer fragments originated by in-flight fragmentation, due to their increased surface area relative to mass, experience greater deceleration from air resistance with respect to the parent bomb, and consequently travel shorter distances^2,69^. The in-flight fragmentation of coarser bombs thus reduces their hazard by reducing their size and hence both their kinetic energy and travel distance. In-flight fragmentation effectively self-limits the ballistic hazard for fluidal bombs during explosive eruptions. Our parameterization of the effect of drag force on in-flight fragmentation provides a quantitative tool to refine ballistic hazard models that already include drag force and collisions but not in-flight fragmentation^4,26,33^.

The potential for in-flight bomb fragmentation to change the grain-size distribution of proximal deposits has been already recognized^5,48,60^, but only partly quantified^12,50^. Our results reveal a non-trivial amount of in-flight fragmentation, averaging 37% and up to 73% of all analysed bombs within the analysed cases and size range. In addition, in-flight fragmentation selectively affects the coarser and faster bombs, with the result that the grain-size distribution of the deposit will be shifted towards finer bombs compared to the erupted one, and increasingly so with increasing distance from the vent.

In the inverse approach, bomb size and distance from the vent have been used as input parameters for ballistic calculations to infer bomb ejection velocity at the vent^7,23,32,34,69^. In-flight fragmentation implies that this approach will overestimate ejection velocity, because some of the coarsest and furthermost bombs measured in the field will not have been emplaced directly along ballistic trajectories but will likely be fragments of coarser and faster bombs. Our in-flight fragmentation criterion based on drag force could potentially be applied also to planetary volcanism, where atmospheric density could be correlated with features of bomb deposits.

## Material and methods

### Video acquisition

For each of the four case studies, we analysed videos of the volcanic activity to describe and quantify the in-flight fragmentation of bombs. Videos were collected with a variety of settings at different distances from the vent (Table 2). The three cameras utilised in this study include a high-speed Optronis CR600X2 camera, a high-definition (4 K) Sony AX100 camcorder, and a high-speed and high-resolution DS-CAM-600 camera. The original monochrome, high-speed, and colour high-definition videos are roughly 20–40 s and minutes to hours in duration, respectively.

**Table 2:** Video acquisition parameters.

Filming date	Volcano	Style of activity	Camera	Frame rate (FPS)	Image resolution (pixels)	Pixel pitch (m/pixel)	Minimum bomb size (automatic tracking)	Error on automatic tracking
22/09/2021	Tajogaite	Fountaining	Optronis	502	1280 × 1024	0.058	0.20–0.13^§^	0.12
29/09/2021	Tajogaite	Spattering	Optronis	500	1280 × 1024	0.056	0.199	0.11
24/02/2021	Etna	Fountaining	Sony	24	3840 × 2160	0.084	0.299	0.17
22/10/2023	Stromboli	Strombolian	DS-CAM-60	250	2048 × 1088	0.02	0.07	0.04

*equivalent diameter of the smallest bomb tracked manually. #equivalent diameter of the smallest bomb tracked by using the semi-automated algorithm. §non-fragmenting manual.

### Video analysis

**Fig. 8:**
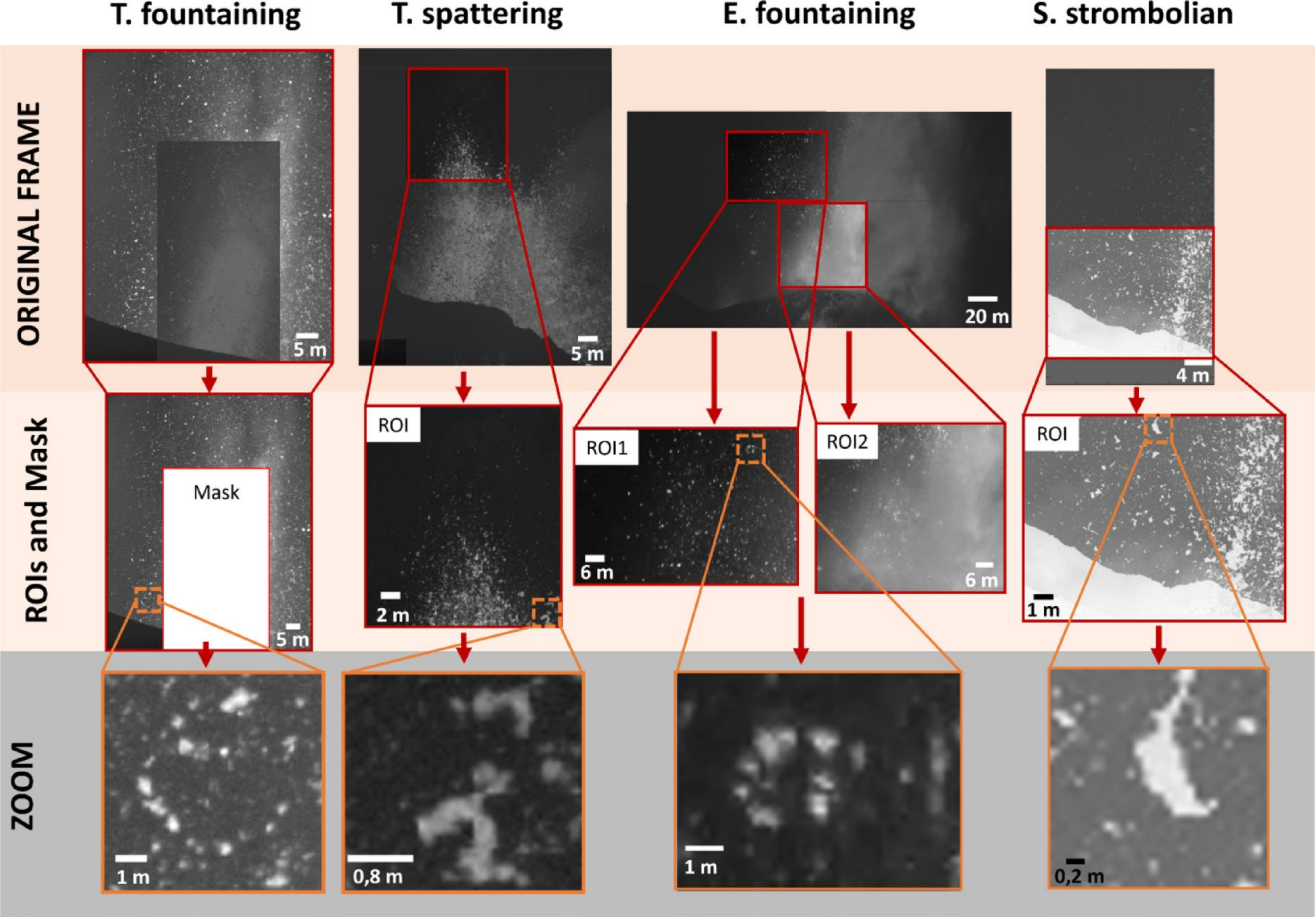
Fig. 8 Still frames from footage of the eruption case studies. In the top row, still frames from the videos with marked the region of interests (ROIs) used to measure bombs. Note that for the T. fountaining case we masked the central, saturated part of the video, while for the E. fountaining case two ROIs were analysed to compare different areas of the fountain. For the E. fountaining and S. strombolian videos, originally in colour, only the red and green channels of the frames have been used, respectively. In the middle row, the analysed ROIs, and in the bottom row, zoomed images on fragmenting bombs. Brighter grey tones indicate hotter bombs in all images.

Within each video we analysed only a specific region of interest (ROI), excluding regions with poor visibility due to, e.g., abundant gas or excessive overlapping of bombs (Fig. 8). In the E. fountaining, two ROIs were defined to investigate differences in the fragmentation in different areas of the fountain. For each video, we analysed only 8–10 s, enough to encompass a whole Strombolian event and most of the variability in the other eruption styles.

In-flight fragmentation of bombs was identified visually in the videos, posing extreme care and running the video backward and forward multiple times to ensure that no bomb overlapping interfered with in-flight fragmentation identification.

Two distinct methodologies were employed to measure the projected (2-D) size, shape, and velocity of bombs.

For the in-flight fragmenting bombs, manual tracking and measuring of hundreds of bombs was performed using the ImageJ freeware^70^ and its MTrackJ plugin^71^. We tracked bombs every ten frames (0.02–0.04 s) for all videos except for the 25 frames per second video of E. fountaining, tracked every frame (0.04 s), found to be suitable by tests at variable frame intervals on the highest frame rate videos. For each tracked bomb we measured the velocity and the area (later converted into equivalent diameter), major and minor axis, aspect ratio (major to minor axis ratio), perimeter, and dimensionless circularity (ranging between 0 and 1 for elongate to circular bombs, respectively) on the most representative frame (i.e., the one showing the larger bomb projection) before fragmentation. The manually tracked bombs are referred to as ‘fragmenting bombs’. For the T. fountaining case, we also manually measured all the 535 non-fragmenting bombs that were observed to transit the ROI in the selected time interval. For the size error of bombs, from repeated measurements we found an error of about ± 1 pixel on equivalent diameter measured from the bomb projected area, thus ranging 0.02–0.08 m from the S. strombolian and the E. fountaining cases, respectively. Pixel pitch poorly affects equivalent diameter (see Supplementary Information). No fixed lower size threshold was determined a priori for the manual tracking and measurement of bombs. The smallest, non-fragmenting bomb manually tracked in the T. fountaining case is 0.13 m in eq. Diameter (Fig. 5), corresponding to 3–4 pixels in area. This is smaller than the smallest fragmenting bomb manually tracked (0.24 m), reassuring us that no significant fraction of fragmenting bombs has been missed due to its small size. The mean standard deviation of the velocity of all tracked bombs ranges from 1.1 m/s in the T. fountaining case to 2.1 m/s for the E. fountaining case. All measured velocities are lower boundaries, because the 2-D projected velocity misses the velocity component towards or away from the camera.

To assess the velocity, size, and shape of all bombs transiting the ROIs in the selected time interval, the semi-automated algorithm of^38^ has been applied to five couples of frames, each couple two-seconds apart, for each of the analysed videos. The five selected couples of frames were pre-processed following^72^ to subtract the background and enhance the definition of bomb boundaries. The pixel-wise displacement field between the two frames in the couple, separated by a time interval function of the video acquisition frame rate, was computed using the OpticalFlowFarneback function in Matlab®^73^. Displacement was then converted into velocity using the time interval between the frames. Subsequently, a variable grey level threshold was applied to each video to delineate bombs from the background, ignoring objects with an area smaller than 10 pixels to remove small artefacts and thus setting the minimum equivalent diameter of the bombs tracked with the algorithm (Table 2). Combined velocity field and analysis of the thresholded frames with the regionprops function of Matlab® provided the velocity, equivalent diameter, major and minor axes, aspect ratio, and circularity of bombs. The effect of variable pixel pitch is minor for the equivalent diameter and relevant on circularity for the smaller bombs (Supplementary Information). A parametric study of algorithm settings on the retrieved parameters is in^38^.The size and velocity distributions of automatically and manually detected bomb populations for the T. fountaining case display a good agreement (Fig. 5, note logarithmic axis scales), the automatically detected population showing a size distribution truncated at the ten pixel threshold and a velocity distribution having a small tail of slow bombs missing in the manually detected population, possibly due to multiple countings of almost static bombs around the top of their trajectories.

The choice of applying the semi-automated algorithm only to five couples of frames for each video was dictated by the need to minimise multiple counting of the same bombs. Considering a ROI with a size of 20–60 m and a mean bomb velocity of 5–20 m/s (Fig. 5), the mean bomb transit time within the ROI ranges about 1–12 s. Given an observation time window of 10 s, the choice of measuring the bombs in five couples of frames two seconds apart represents a balance between an overestimate of the slowest and an underestimate of the fastest bombs. The bombs measured with the semi-automated algorithm are referred to as ‘total bombs’. To account for the variability in the number of bombs visible at different times, the results of the five couples of frames were then averaged to obtain the total number of bombs. Due to uncertainties in the number of bombs obtained by the algorithm, mostly function of the chosen threshold setting and the pulsatory nature of the activity (see Supplementary Methods in^38^), the algorithm results were compared against the total number of bombs measured manually for the T. fountaining case, assumed to be more accurate. The manually tracked bombs are 62% of the averaged automatic detections in the size range of the fragmenting bombs, the difference accounting for residual double counting and other bomb misidentifications of the algorithm. Extending this result to all videos, the total number of transiting bombs in all cases was obtained by multiplying by 0.62 the average number of bombs automatically detected over the five couples of frames. To be as conservative as possible, the total and the fragmenting bombs populations were compared taking into account only bombs equal to or larger than the smallest fragmenting bombs (Fig. 5). Alternate estimates were also obtained by comparing the fragmenting bomb populations to the total bomb populations obtained in different ways with and without normalization by the manually tracked total population (see Supplementary Information).

## Supplementary Information

Below is the link to the electronic supplementary material.

Supplementary Material 1

Supplementary Material 2

Supplementary Material 3

Supplementary Material 4

Supplementary Material 5

Supplementary Material 6

Supplementary Material 7

Supplementary Material 8

Supplementary Material 9

Supplementary Material 10

Supplementary Material 11

Supplementary Material 12

Supplementary Material 13

Supplementary Material 14

Supplementary Material 15

Supplementary Material 16

Supplementary Material 17

Supplementary Material 18

Supplementary Material 19

Supplementary Material 20

Supplementary Material 21

Supplementary Material 22

Supplementary Material 23

Supplementary Material 24

Supplementary Material 25

Supplementary Material 26

Supplementary Material 27

## Data Availability

The datasets generated and analysed during the current study are available in the Zenodo repository, 10.5281/zenodo.14245877.
