# Supplementary material for: In flight fragmentation reduces bomb size range and hazard during explosive volcanic eruptions
Source: Sci Rep. 2025 Oct 22;15:36880. doi: 10.1038/s41598-025-20900-2 (PMC12546931; doi:10.1038/s41598-025-20900-2)
Supplement: Supplementary file 26 — Supplementary Material 26 [file 41598_2025_20900_MOESM26_ESM.pdf]

Supplementary Information for

In-flight fragmentation reduces the size, range, and hazard of bombs from explosive volcanic eruptions

C. Biensan (1,6), J. Taddeucci (1), M. Alatorre-Ibarguengoitia (2), P. Scarlato (1), D. Andronico (3), T. Ricci (1), E. Del Bello (1), L. D'Auria (4,5), M. Asensio-Ramos (4), D.M. Palladino (6)

(1) Istituto Nazionale di Geofisica e Vulcanologia, Sezione Roma 1, Via di Vigna Murata 605, 00143 Roma, Italy

(2) Instituto de Investigación en Gestión de Riesgo y Cambio Climático, Universidad de Ciencias y Artes de Chiapas, Libramiento Norte poniente 1150, Lajas Maciel, Tuxtla Gutiérrez, 29039 Tuxtla Gutiérrez, Chiapas, México

(3) Istituto Nazionale di Geofisica e Vulcanologia, Osservatorio Etneo, Piazza Roma 2, 95125, Catania, Italy

(4) Instituto Tecnológico y de Energías Renovables (ITER), 38600 Granadilla de Abona, Tenerife, Canary Islands, Spain

(5) Instituto Volcanológico de Canarias (INVOLCAN), 38320 San Cristóbal de La Laguna, Tenerife, Canary Islands, Spain

(6) Sapienza-Università di Roma, Dipartimento di Scienze della Terra, Piazzale Aldo Moro 5, 00185, Rome, Italy

Description and methods for the Supplementary Movies.

Supplementary Movies 1 to 4 are representative of the original videos of the four eruption case studies. Supplementary Movies 5 to 25 exemplify the different modes of in-flight fragmentation of volcanic bombs from the four case studies. These videos were generated by processing manual tracks of fragmenting bombs to produce cropped, zoomed video sequences that highlight the fragmentation process. We interpolated positions between tracked points and for each point cropped, from the original high-resolution frame, a small area of 61 by 61 pixels (except Movie 21 with an area of 131 by 131 pixels) centred around the bomb. The cropped images were saved and merged together into videos with a playback frame rate between 20 and 27 frames per second (FPS). For Movies 1 to 4 we reduced the original length and resolution to decrease the final file size, adjusted the final playback rate between 20 and 50 FPS. Movie 21 has been resampled at 1000 x 1000 pixels. Movies 1-4 and 21 were compressed in MOV format.

Dimensionless Characterization.

Following recent literature (Edwards et al., 2021; Comida et al., 2024), we performed a dimensionless characterization of our data, in order to generalize the results to other volcanic and non-volcanic cases of in-flight fragmentation. We calculated the Reynolds (Re) and Weber (We) dimensionless groups, respectively representing the ratio of the inertial to viscous and inertial to capillary forces, also combining them into the Ohnesorge (Oh) group. These are:

$$Re = \frac{\rho V D}{\mu} \quad 1)$$

$$We = \frac{\rho V^2 D}{\sigma} \quad 2)$$

$$Oh = \frac{\sqrt{We}}{Re} \quad 3)$$

where *V*, *D*, *r*, and *m*, are the bomb velocity, diameter, density, and viscosity and s is the bomb-air surface tension. *V* and *D* are obtained from our video analysis; we used 0.3 for σ for all cases, in line with Comida et al. (2024); for *r* we used 1000, 1000, 1250 and 1500 kg/m^3 for T. fountaining and T. spattering (Romero et al., 2022)., E. fountaining (Polacci et al., 2006), and S. strombolian (Bonbrum et al., 2015), respectively; and for μ we used 400, 400, 2500, and 3000 Pa s for T. fountaining and T. spattering (Gisbert et al., 2023)., E. fountaining (Taddeucci et al., 2004), and S. strombolian (Gurioli et al., 2013) , respectively.

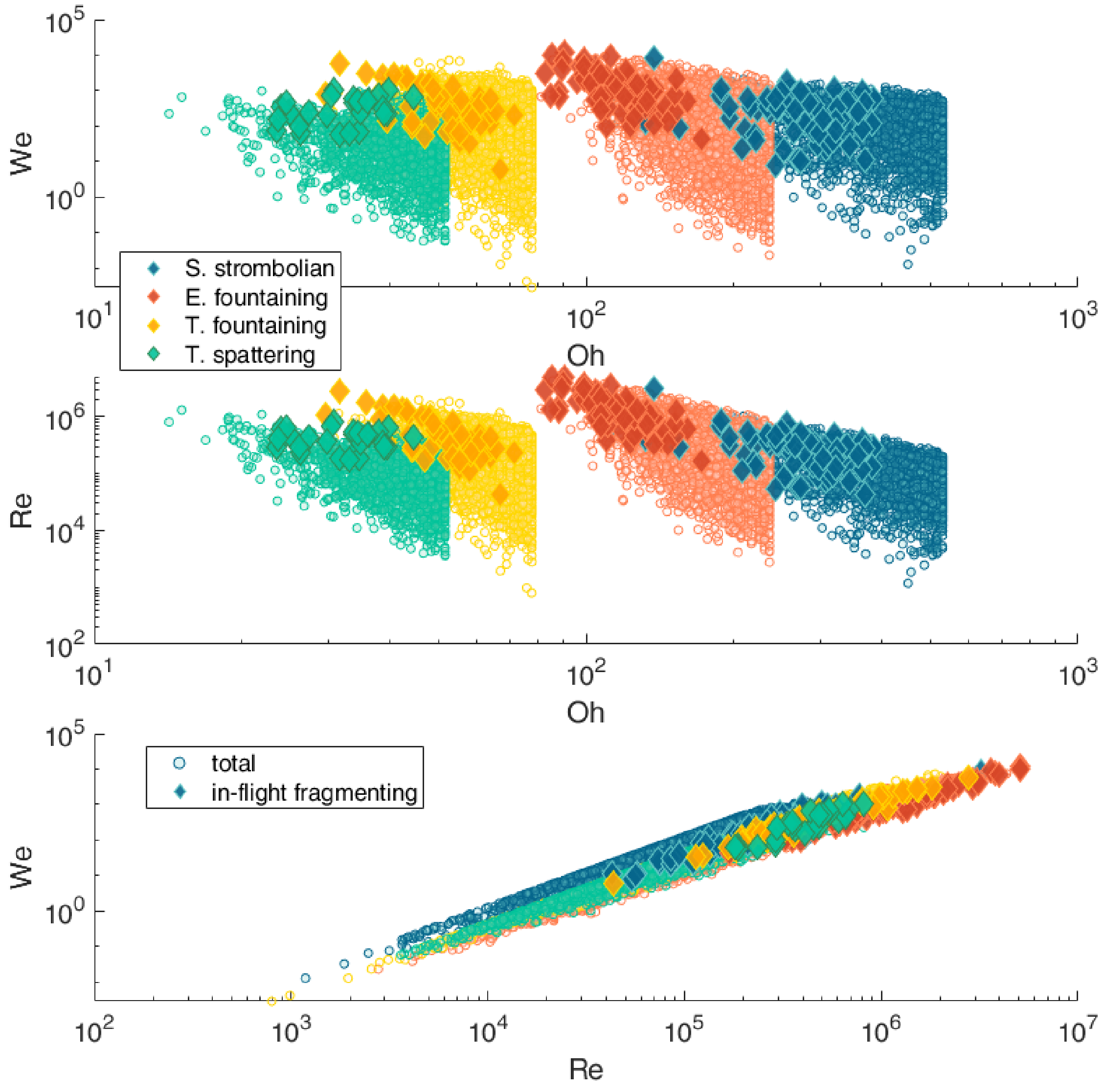

Fig.-S1: Dimensionless Weber (We), Ohnesorge (Oh), and Reynolds (Re) groups for the in-flight fragmenting and all bomb populations.

In-flight fragmenting bombs are well divided from non-fragmenting ones in the Re-We field (Fig. S1), both parameters mainly depending on *D* and *V*. Of the other chosen parameters, viscosity divides the four cases for Oh, but does not seem to have a clear effect on the in-flight fragmentation. Considering all uncertainties and assumptions on the choice of the input parameters, the results support the strong control of bomb size and velocity, hence drag force (Fd), on the in-flight fragmentation. They also support our conclusion that, more than the initial properties of the magma at eruption, it is the dynamically-changing properties acquired during in-flight that control the in-flight fragmentation of bombs.

Pixelization.

Digital rendering (pixelization) and variable pixel pitch in the different videos affect bomb measurements. To explore these effects, we took from the S. strombolian video the digital images of two bombs with different shape and size and resampled them with different pixel size, effectively mimicking videos acquired at different resolutions. On these, we measured

the perimeter and the circularity of the original and the resampled images with both the ImageJ software and with the Matlab® 'regionprops' function, used for the manual and the semi-automatic measurements, respectively.

	Pixel pitch (a.u.)	Area (pixel)	Eq. Diam. (a.u.)	Perim. (pixel)	Circ. (adim.)
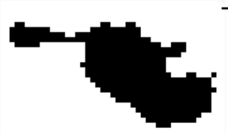	1	372	21.8	122.1 J<br>115.9 R	0.313 J<br>0.330 R
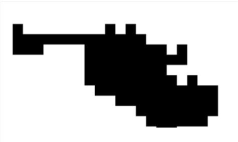	2	94	21.9	59.6 J<br>56.1 R	0.333 J<br>0.337 R
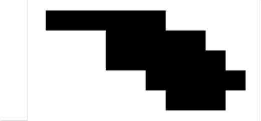	4	25	22.6	23.6 J<br>20.8 R	0.566 J<br>0.549 R
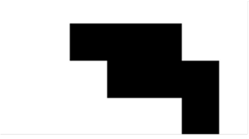	8	7	23.9	10.5 J<br>7.6 R	0.800 J<br>0.764 R
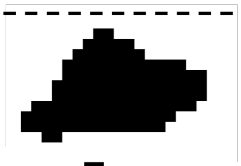	1	117	12.2	45.7 J<br>42.4 R	0.704 J<br>0.701 R
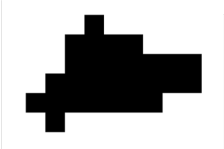	2	28	11.9	23.0 J<br>20.1 R	0.667 J<br>0.649 R
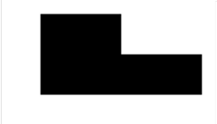	4	6	11.1	9.1 J<br>6.9 R	0.916 J<br>0.745 R

Fig.-S2: Effect of pixel pitch on image analysis of bombs. On the left-hand, the images of two bombs at the original resolution and at different relative pixel pitches, obtained by resampling the images at different ratios to the original (column 'pixel size'). The equivalent diameter was calculated from the area accounting for the relative pixel pitch. The perimeter and the circularity values are marked 'J' and 'R' if measured with ImageJ and with Matlab® 'regionprops' function, respectively.

The computed equivalent diameter increases or decreases with increasing pixel pitch depending on bomb shape, but overall the effect is minor, with an eight-fold increase in pixel pitch causing a <10% change in the equivalent diameter. Conversely, circularity increases nonlinearly with increasing pixel pitch, with a sharp increase when the number of pixels in the bomb approaches our threshold of 10 pixels (Fig. S2). This effect is more relevant for the manually tracked bombs that were analyzed with ImageJ, because the two softwares use slightly different methods for perimeter and circularity measurements.

Number of bombs and percent of fragmenting bombs: alternative methods.

As described in the main text, we estimated the total number of bombs by using a semiautomated algorithm to measure the number of bombs occurring in five couples of frames two seconds apart. To compare the total number of bombs with the number of inflight fragmenting bombs for each case study, first we discarded all bombs smaller than the smallest fragmenting bomb, and then we normalized the number of remaining total bombs by multiplying them by the ratio of manually detected bombs to total bombs for the F. fountaining case. Below, we report the total number of bombs and the fragmenting bomb percentage obtained by using alternative processing methods of the data obtained by the semi-automated algorithm.

In the table below the following nomenclature applies:

1) total: the sum of all bombs counted in the five couples of frames analyzed.

2) total GIAC.: a method, kindly suggested by Thomas Giachetti, that normalizes the total results taking into account the vertical velocity of bombs (bomb_speed), ROI vertical size (ROI_size), and the time between couples (2 s) to weight the number of detected bombs, assuming a constant vertical velocity.

SHEA total=sum(2/(ROI_size / bomb_speed))

3) average: total divided by the number of couples (5).

4) size filtered: all bombs minus bombs smaller than the smallest fragmenting bomb (per each case).

5) T. fount. norm. factor: the number of all bombs manually detected for the T. fountaining case divided by the number of bombs obtained for the same case with the different data processing methods (total, total GIAC. average), for the size filtered and not size filtered cases.

**manually detected bombs (#)**			
	total	total size filtered	fragmenting
T. fountaining	595	490	60
T. spattering			102
E. fountaining ROI 1			88
E. fountaining ROI 2			56
S. strombolian			65

**automatically detected bombs (#)**						
	total	total size filtered	total GIAC.	total GIAC. size filtered	average	average size filtered
T. fountaining	5708	3968	2306	1524	1142	794
T. spattering	2057	1129	821	455	411	226
E. fountaining ROI 1	3541	1610	2078	758	708	322
E. fountaining ROI 2	4762	1392	1912	311	952	278
S. strombolian	2879	985	4943	1487	576	197

**fragmenting bombs (%)**						
	total	total size filtered	total GIAC.	total GIAC. size filtered	average	average size filtered
T. fountaining	1.1	1.5	2.6	3.9	5.3	7.6
T. spattering	5.0	9.0	12.4	22.4	24.8	45.2
E. fountaining ROI 1	2.5	5.5	4.2	11.6	12.4	27.3
E. fountaining ROI 2	1.2	4.0	2.9	18.0	5.9	20.1
S. strombolian	2.3	6.6	1.3	4.4	11.3	33.0

**fragmenting bombs with normalization to manual tracking of T. fountaining (%)**						
	total	total size filtered	total GIAC.	total GIAC. size filtered	average	average size filtered
T. fount. norm. factor	0.10	0.12	0.26	0.32	0.52	0.62
T. fountaining	10.1	12.2	10.1	12.2	10.1	12.2
T. spattering	47.6	73.2	48.2	69.7	47.6	73.2
E. fountaining ROI 1	23.8	44.3	16.4	36.1	23.8	44.3
E. fountaining ROI 2	11.3	32.6	11.4	56.0	11.3	32.6
S. strombolian	21.7	53.4	5.1	13.6	21.7	53.4

Given uncertainties in: i) the assumptions intrinsic in the different data processing methods, and 2) the manual detection of small fragmenting and non-fragmenting bombs, we included in the main text the size filtered normalized results, considered to be the most self-consistent and conservative ones.

Supplementary Information references.

Comida, P. P., & Jones, T. J. (2024). Spread or splash: The ubiquitous role of droplets in mafic explosive eruptions. Geochemistry, Geophysics, Geosystems, 25, e2024GC011770. https://doi.org/10.1029/2024GC011770

Romero, J. E., Burton, M., Cáceres, F., Taddeucci, J., Civico, R., Ricci, T., ... & Perez, N. M. (2022). The initial phase of the 2021 Cumbre Vieja ridge eruption (Canary Islands): Products and dynamics controlling edifice growth and collapse. Journal of Volcanology and Geothermal Research, 431, 107642.

Polacci, M., Corsaro, R. A., & Andronico, D. (2006). Coupled textural and compositional characterization of basaltic scoria: Insights into the transition from Strombolian to fire fountain activity at Mount Etna, Italy. Geology, 34(3), 201-204.

Gisbert, G., Troll, V. R., Day, J. M., Geiger, H., Perez-Torrado, F. J., Aulinas, M., ... & Carracedo, J. C. (2023). Reported ultra-low lava viscosities from the 2021 La Palma eruption are potentially biased. Nature Communications, 14(1), 6453.

Gurioli, L., Harris, A. J. L., Colò, L., Bernard, J., Favalli, M., Ripepe, M., & Andronico, D. (2013). Classification, landing distribution, and associated flight parameters for a bomb field emplaced during a single major explosion at Stromboli, Italy. Geology, 41(5), 559-562.

Edwards, M. J., Pioli, L., Harris, A. J., Gurioli, L., & Thivet, S. (2020). Magma fragmentation and particle size distributions in low intensity mafic explosions: the July/August 2015 Piton de la Fournaise eruption. Scientific reports, 10(1), 13953.

Taddeucci, J., Pompilio, M., & Scarlato, P. (2004). Conduit processes during the July–August 2001 explosive activity of Mt. Etna (Italy): inferences from glass chemistry and crystal size distribution of ash particles. Journal of Volcanology and Geothermal Research, 137(1-3), 33-54.
