# Supplementary material for: In flight fragmentation reduces bomb size range and hazard during explosive volcanic eruptions
Source: Sci Rep. 2025 Oct 22;15:36880. doi: 10.1038/s41598-025-20900-2 (PMC12546931; doi:10.1038/s41598-025-20900-2)
Supplement: Supplementary file 27 — Supplementary Material 27 [file 41598_2025_20900_MOESM27_ESM.docx]

**In flight fragmentation reduces bomb size range and hazard during explosive volcanic eruptions**

*C. Biensan (1,6), J. Taddeucci (1), M. Alatorre-Ibarguengoitia (2), P. Scarlato (1), D. Andronico (3), T. Ricci (1), E. Del Bello (1), L. D’Auria (4,5), M. Asensio-Ramos (4), D.M. Palladino (6)*

(1) Istituto Nazionale di Geofisica e Vulcanologia, Sezione Roma 1, Via di Vigna Murata 605, 00143 Roma, Italy

(2) Instituto de Investigación en Gestión de Riesgo y Cambio Climático, Universidad de Ciencias y Artes de Chiapas, Libramiento Norte poniente 1150, Lajas Maciel, Tuxtla Gutiérrez, 29039 Tuxtla Gutiérrez, Chiapas, México

(3) Istituto Nazionale di Geofisica e Vulcanologia, Osservatorio Etneo, Piazza Roma 2, 95125, Catania, Italy

(4) Instituto Tecnológico y de Energías Renovables (ITER), 38600 Granadilla de Abona, Tenerife, Canary Islands, Spain

(5) Instituto Volcanológico de Canarias (INVOLCAN), 38320 San Cristóbal de La Laguna, Tenerife, Canary Islands, Spain

(6) Sapienza-Università di Roma, Dipartimento di Scienze della Terra, Piazzale Aldo Moro 5, 00185, Rome, Italy

**Descripfion of Addifional Supplementary Files.**

**Supplementary Movie 1:** **Tajogaite fountaining activity.**
The video has a horizontal field view of 74 m, with a duration of 2 seconds in nature.
The video shows fountaining activity during the 22 September 2021, Tajogaite eruption of the Cumbre Vieja volcano, La Palma, Canary Islands, Spain.

**Supplementary Movie 2:** **Stromboli strombolian activity.**
The video has a horizontal field view of 22 m, with a duration of 2 seconds in nature.
The video shows strombolian activity observed on 22 October 2023, at Stromboli volcano, Aeolian Islands, Italy.

**Supplementary Movie 3: Mount Etna fountaining activity.**
The video has a horizontal field view of 323 m, with a duration of 2 seconds in nature.
The video shows fountaining activity during the 24 February 2021 eruption of Mount Etna, Sicily, Italy.

**Supplementary Movie 4: Tajogaite spattering activity.**
The video has a horizontal field view of 72 m, with a duration of 2 seconds in nature.
The video shows spattering activity during the 29 September 2021, Tajogaite eruption of the Cumbre Vieja volcano, La Palma, Canary Islands, Spain.

**Supplementary Movie 5: Etna fountaining fragmenting bomb under the stretching mode.**The video has a horizontal field view of 5.12 m, with a duration of 1.3 seconds in nature.
The video focuses on a bomb fragmenting progressively as it stretches while falling. In the background, bombs are falling at different velocities.

**Supplementary Movie 6: Stromboli strombolian fragmenting bomb under the stretching mode.**The video has a horizontal field view of 1.22 m, with a duration of 0.23 seconds in nature.
The video focuses on a rising bomb fragmenting while stretching at its thinner point. In the background, bombs are falling and rising at different velocities.

**Supplementary Movie 7: Tajogaite fountaining fragmenting bomb under the stretching mode.**The video has a horizontal field view of 3.54 m, with a duration of 2.32 seconds in nature.
The video focuses on a coarse, falling bomb fragmenting progressively as it stretches and becomes thinner. In the background, numerous bombs are falling and rising at different velocities.

**Supplementary Movie 8: Tajogaite spattering fragmenting bomb under the stretching mode.**The video has a horizontal field view of 3.42 m, with a duration of 0.38 seconds in nature.
The video focuses on a coarse, rising bomb with two larger extremities. The middle section becomes increasingly thinner, fragmenting when the connection weakens. In the background, numerous bombs are falling, with few rising at different velocities.

**Supplementary Movie 9: Etna fountaining fragmenting bomb under the bending mode.**The video has a horizontal field view of 5.12 m, with a duration of 1.2 seconds in nature.
The video is centred on a bomb fragmenting through bending while rising. In the background, other bombs are rising and falling.

**Supplementary Movie 10: Stromboli strombolian fragmenting bomb under the bending mode.**The video has a horizontal field view of 1.22 m, with a duration of 0.28 seconds in nature.
The video focuses on a rising bomb whose lower part is bending from right to left until fragmentation. In the background, bombs are falling at different velocities.

**Supplementary Movie 11: Tajogaite fountaining fragmenting bomb under the bending mode.**The video has a horizontal field view of 3.54 m, with a duration of 2.32 seconds in nature.
The video focuses on a thin, falling bomb fragmenting while bending at its center. In the background, numerous small bombs are falling and rising at different velocities.

**Supplementary Movie 12:** **Tajogaite spattering fragmenting bomb under the bending mode.**The video has a horizontal field view of 3.42 m, with a duration of 0.34 seconds in nature.
The video focuses on a falling bomb fragmenting as it bends at its thinner point, causing a circular piece to detach from a long, thin section. In the background, numerous bombs are falling, with few rising at different velocities.

**Supplementary Movie 13: Etna fountaining fragmenting bomb under the rotating mode.**The video has a horizontal field view of 5.12 m, with a duration of 0.9 seconds in nature.
The video focuses on a bomb that first rises and then falls, fragmenting while rotating during this transition. In the background, bombs are falling at different velocities.

**Supplementary Movie 14: Stromboli strombolian fragmenting bomb under the rotating mode.**The video has a horizontal field view of 1.22 m, with a duration of 1.56 seconds in nature.
The video focuses on a thin bomb that rises and then falls, fragmenting while rotating at its thinner point. In the background, bombs are falling and rising at different velocities.

**Supplementary Movie 15: Tajogaite fountaining fragmenting bomb under the rotating mode.**The video has a horizontal field view of 3.54 m, with a duration of 2.17 seconds in nature.
The video focuses on a falling bomb fragmenting at its thinner point while rotating . In the background, numerous bombs are falling and rising at different velocities.

**Supplementary Movie 16: Tajogaite spattering fragmenting bomb under the rotating mode.**The video has a horizontal field view of 3.42 m, with a duration of 0.44 seconds in nature.
The video focuses on a falling bomb fragmenting at its thinner point while rotating. In the background, numerous bombs are falling, with few rising at different velocities.

**Supplementary Movie 17:** **Etna fountaining fragmenting bomb under the detaching mode.**The video has a horizontal field view of 5.12 m, with a duration of 1.4 seconds in nature.
The video focuses on a falling bomb fragmenting in three pieces without any visible deformation.

**Supplementary Movie 18: Stromboli strombolian fragmenting bomb under the detaching mode.**The video has a horizontal field view of 1.22 m, with a duration of 0.13 seconds in nature.
The video focuses on a falling bomb breaking through detachment, with finer sections detaching from coarser parts. In the background, bombs are falling and rising at different velocities.

**Supplementary Movie 19: Tajogaite fountaining fragmenting bomb under the detaching mode.**The video has a horizontal field view of 3.54 m, with a duration of 3.00 seconds in nature.
The video focuses on a coarse, bright (hot), falling bomb with a darker (colder) section detaching from it. In the background, numerous bombs are falling and rising at different velocities.

**Supplementary Movie 20: Etna fountaining fragmenting bomb under the inflation mode.**The video has a horizontal field view of 5.12 m, with a duration of 1.4 seconds in nature.
The video focuses on a rising bomb fragmenting while inflating. Once it breaks, the bomb begins rotating due to its new shape. In the background, bombs are falling.

**Supplementary Movie 21: Tajogaite fountaining fragmenting bomb under the inflation mode.**The video has a horizontal field view of 7.6 m, with a duration of 2.8 seconds in nature.
The video focuses on a very coarse falling bomb. Early in the video, the bomb is slowly deforming and displays variable grey levels that reflect variable surface temperature. An impact with a rising bomb (at the beginning of second two of the video) causes fragmentation and triggers generalized inflation and fragmentation of the coarse bomb, with breaking of the colder crust and exposure of the hotter interior (brighter grey tones). In the rest of the video, the bomb surface gradually cools down. In the background, numerous lighter-colored bombs are rising while darker ones are falling.

**Supplementary Movie 22: Etna fountaining fragmenting bomb under the collision mode.**The video has a horizontal field view of 5.12 m, with a duration of 1.2 seconds in nature.
The video focuses on a rising bomb that first fragments while stretching. In a subsequent stage, a falling bomb collides with it, producing numerous (at least 10) fragments. In the background, other bombs are rising and falling.

**Supplementary Movie 23:** **Stromboli strombolian fragmenting bomb under the collision mode.**The video has a horizontal field view of 1.22 m, with a duration of 0.98 seconds in nature.
The video focuses on a rising bomb colliding with a falling one. The stretched section of the falling bomb encounters the coarser, rising bomb, resulting in stretching and fragmentation. In the background, bombs are falling and rising at different velocities.

**Supplementary Movie 24:** **Tajogaite fountaining fragmenting bomb under the collision mode.**The video has a horizontal field view of 3.54 m, with a duration of 0.52 seconds in nature.
The video focuses on a coarse, falling bomb colliding with a finer, rising bomb, causing the coarse bomb to fragment into at least six pieces. In the background, numerous small bombs are falling and rising at different velocities.

**Supplementary Movie 25: Tajogaite spattering fragmenting bomb under the collision mode.**The video has a horizontal field view of 3.42 m, with a duration of 1.22 seconds in nature.
The video focuses on a coarser, falling bomb colliding with two finer, rising bombs, causing the coarser bomb to fragment into numerous small pieces. In the background, numerous bombs are falling, with few rising at different velocities.
